# Cystathionine β-synthase-derived hydrogen sulfide regulates lipopolysaccharide-induced apoptosis of the BRL rat hepatic cell line *in vitro*

**DOI:** 10.3892/etm.2012.672

**Published:** 2012-08-16

**Authors:** JUN YAN, FEIXIANG TENG, WEIWEI CHEN, YINGLEI JI, ZHENYONG GU

**Affiliations:** 1 Department of Forensic Medicine, Soochow University, Jiangsu 215123;; 2 Department of Physiology, Yancheng Institute of Health Science, Jiangsu 224000;; 3 Department of Anorectal Surgery, Yancheng Third People’s Hospital, Jiangsu 224001, P.R. China

**Keywords:** cystathionine β-synthase, hydrogen sulfide, apoptosis, lipopolysaccharide, BRL

## Abstract

Hydrogen sulfide (H_2_S), is a member of the novel family of endogenous gaseous transmitters, termed “gasotransmitters exhibiting diverse physiological activities, and is generated in mammalian tissues mainly by cystathionine β-synthase (CBS), cystathionine γ-lyase (CSE) and 3-mercaptopyruvate sulfurtransferase (3MST) in conjunction with cysteine (aspartate) aminotranferase (CAT). The distributions of these enzymes are species- and tissue-specific. The liver, as the main organ that generates H_2_S *in vivo*, functions in biotransformation and metabolism. However, the liver is vulnerable to damage from internal and external factors, including inflammatory mediators, drugs and poisons. The present study evaluated the endogenous CBS-H_2_S synthesis regulating lipopolysaccharide (LPS)-induced apoptosis of hepatic cells. The rat hepatic cell line, BRL, was incubated with LPS for various time periods to establish a cell-damage model. Incubation with LPS resulted in a significant increase in CBS expression and H_2_S production. It also stimulated apoptosis and decreased the mitochondrial membrane potential. Pretreatment with the CBS inhibitor aminooxyacetic acid (AOAA) or CBS small interfering RNA (siRNA) decreased LPS-enhanced H_2_S production. Notably, apoptosis increased for a short period and then decreased gradually, while the mitochondrial membrane potential demonstrated the opposite trend. These results showed that endogenous CBS-H_2_S synthesis demonstrated early anti-apoptotic activity and subsequent pro-apoptotic activity in LPS-induced apoptosis. These results suggest a new approach for developing novel drugs for this condition.

## Introduction

Hydrogen sulfide (H_2_S), a novel and important gaseous transmitter, is endogenously generated in mammalian tissues predominantly by two pyridoxal-5′-phosphate-dependent enzymes, cystathionine β-synthase (CBS) and cystathio-nine γ-lyase (CSE), using either L-cysteine or L-homocysteine as a substrate (1,2). Recently, Shibuya *et al* (3) observed that 3-mercaptopyruvate sulfurtransferase (3MST) in conjunction with cysteine (aspartate) aminotranferase (CAT), as the third H_2_S-producing enzyme, contributes significantly to generating H_2_S from L-cysteine in the presence of α-ketoglutarate. The distribution of these enzymes is species- and tissue-specific, and CBS and CSE are present in the liver (4). H_2_S has been observed to demonstrate a wide range of physiological functions and is important for several pathological conditions. For example, H_2_S opens K^+^-ATP channels in vascular and gastrointestinal smooth muscle cells, neurons and pancreatic β cells, regulating vascular tone, intestinal contractility, neurotransmission and insulin secretion (5–8). H_2_S has also been recognized to be involved in the inflammatory response (9).

Bacterial endotoxins such as lipopolysaccharide (LPS) induce excessive activation and upregulation of vascular K^+^-ATP channels and hypotension, and substantially reduce vascular sensitivity to vasoconstrictive agents (10,11). H_2_S has been proposed as a potential endogenous ligand for K^+^-ATP channels to induce K^+^-ATP channel-mediated vasorelaxation (12) in several vascular tissues, suggesting that H_2_S might be involved in endotoxic shock.

As one of the most important organs in the body, the liver functions in biotransformation and metabolism. Due to its central location in regulating metabolism and response to both physiological and pathological exogenous stimuli, all forms of liver disease are accompanied by a certain degree of inflammation. Therefore, the liver is vulnerable to damage from internal and external factors, including inflammatory mediators, drugs and poisons (13,14). Numerous studies have investigated the overall function and mechanism of this organ and in related research, the role of hydrogen sulfide in hepatic ischemia-reperfusion injury has been reported (15). However, the significance of endogenous H_2_S (particularly the CBS-H2S synthesis) in endotoxemia at the hepatic cell level is rarely discussed.

In the present study, we used the BRL rat hepatic cell line to imitate hepatocytes *in vivo*. We hypothesized that the endogenous CBS-H_2_S synthesis participates in the pathophysiological regulation of apoptosis induced by LPS. To investigate the role of the CBS-H_2_S synthesis in the pathogenesis of hepatocyte injury, we added LPS to BRL cells and observed changes in endogenous CBS expression, H_2_S concentration in the culture supernatant and regulation of apoptosis following administration of CBS inhibitor or CBS small interfering RNA (siRNA).

## Materials and methods

### Materials

LPS (*Escherichia coli* 0111:B4) and aminooxyacetic acid (AOAA) were obtained from Sigma-Aldrich (St. Louis, MO, USA). Other chemicals and reagents were of analytical grade.

### Cell culture

The normal rat hepatic cell line BRL was obtained from the Cell Bank of the Chinese Academy of Sciences, Shanghai, China. BRL cells were cultured at 37°C in a humidified incubator with 95% air and 5% CO_2_ in RPMI-1640 medium (Gibco, Invitrogen, Carlsbad, CA, USA) supplemented with 10% heat-inactivated fetal bovine serum (FBS; Hyclone, Invitrogen), 100 U/ml of penicillin and 100 μg/ml of streptomycin. Cultured cells were used at 70–80% confluence.

### siRNA

CBS siRNA sequences were designed and synthetized by Invitrogen: siRNA 455-s, 5′-CCAAGUGUGAGUUCUUCAATT-3′ and siRNA 455-a, 5′-UUGAAGAACUCACACU UGGTT-3′; siRNA 1283-s, 5′-CCAAGUUCUUGAGUGACAATT-3′ and siRNA 1283-a, 5′-UUGUCACUCAAGAACUUGGTT-3′; siRNA 1739-s, 5′-CCAUUGACCUGCUAAACUUTT-3′ and siRNA 1739-a, 5′-AAGUUUAGCAGGUCAAUGGTT-3′. One OD unit of each siRNA was dissolved in 150 μl double-distilled H_2_O and 18 μl was mixed with 6 μl Lipofectamine™ 2000 Transfection Reagent (Invitrogen) in 550 μl serum-free medium for 30 min. This was added to cells in 6-well plates. Western blot analysis was used to detect the efficiency of gene silencing. The most efficient sequence was used to transfect cells for flow cytometry (FCM) dectection.

### CBS mRNA assay

Total RNA from BRL cells was extracted using TRIzol reagent (Gibco, Invitrogen). Reverse transcription-polymerase chain reaction (RT-PCR) was performed in a 0.2-ml tube containing 2 μl tissue cDNA, 1 μl primer mixture of 5 μmol/l of each CBS-s, 5′-GAACCAGACGGAGCAAACAG-3′ and CBS-a, 5′-TGTAGAGGACTTTGCAGACT-3′ (Invitrogen), 1 μl of 2.5 mmol/l each dNTP, 1.5 μl of 1.5 mmol/l MgCl_2_, 2.5 μl 10X PCR buffer and 1.25 U Taq DNA polymerase, in 25 μl. After incubation at 95°C for 5 min, PCR was performed at 94°C for 30 sec, 55°C for 30 sec and 72°C for 40 sec for 30 cycles. The PCR products were separated on a 1.5% agarose gel and stained with ethidium bromide. The optical density of the band of CBS mRNA (572 bp) was measured using the Gel Documentation System (Bio-Rad, Hercules, CA, USA). PCR products were amplified again at 94°C for 30 sec, 55°C for 30 sec and 72°C for 30 sec for 20 cycles with rat GAPDH primers: GAPDH-s, 5′-C CATGACAACTTTGGCATC-3′ and GAPDH-a, 5′-ATGTCA GATCCACAACGGA-3′ (Invitrogen). The optical density of the GAPDH mRNA band (262 bp) was measured and the ratio of CBS mRNA/GAPDH mRNA was taken to be the relative quantity of CBS mRNA.

### Preparation of cell lysates for western blot analysis

After treatment, BRL cells were homogenized in ProteoJET™ mammalian cell lysis reagent supplemented with ProteoBlock™ protease inhibitor cocktail (Fermentas, Amsterdam, The Netherlands) and centrifuged at 4°C for 15 min at 16,000 × g. The supernatants were collected and stored at −80°C. Protein concentrations were determined by the Bio-Rad protein assay (Bio-Rad).

### Western blot analysis

The protein samples (30 μg) were separated by SDS-polyacrylamide gel electrophoresis on 10% Tris-glycine polyacrylamide gels and transferred to nitrocellulose membranes. Nonspecific binding was blocked by incubation for 1 h in 5% nonfat dry milk in PBST (0.05% Tween-20 in phosphate-buffered saline). The blots were incubated overnight with primary antibody against CBS, cytochrome *c*, or cleaved caspase-3 (Asp175; Santa Cruz Biotechnology, Inc., Santa Cruz, CA, USA) at 1:400 in 2.5% nonfat dry milk in PBST, followed by washing 4 times with PBST and incubating for 1 h with goat anti-rabbit horseradish peroxidase-conjugated secondary antibody (Santa Cruz Biotechnology, Inc.) at 1:2,000 in 2.5% nonfat dry milk in PBST. The membranes were washed and incubated in SuperSignal West Pico chemiluminescent substrate (Pierce Chemical, Rockford, IL, USA) before exposure to X-ray film (CL-XPosure; Pierce Chemical). The gels were calibrated by protein Kaleidoscope standards (Bio-Rad). β-tubulin (Santa Cruz Biotechnology, Inc.) was used as an internal control to normalize for protein loading. Band intensity was quantified using LabWorks Image Analysis software (UVP Upland, CA, USA).

### Measurement of H_2_S production

H_2_S production was measured as described previously (12,16). Briefly, after treatment, cells were collected and homogenized in 50 mM ice-cold potassium phosphate buffer (pH 6.8). Flasks containing reaction mixture (100 mM potassium phosphate buffer, 10 mM L-cysteine, 2 mM pyridoxal 5′-phosphate and 10% w/v cell homogenates) and center wells containing 0.5 ml 1% zinc acetate and a piece of filter paper were flushed with N_2_ and incubated at 37°C for 90 min. The reaction was terminated by adding 0.5 ml 50% trichloroacetic acid and flasks were incubated at 37°C for 60 min. The contents of the center wells were transferred to test tubes each containing 3.5 ml of water and 0.5 ml of 20 mM N, N-dimethyl-p-phenylenediamine sulfate in 7.2 M HCl and 0.5 ml 30 mM FeCl_3_ in 1.2 M HCl were added. The absorbance of the resulting solution at 670 nm was measured after 20 min with a Multiskan^®^ spectrum microplate spectrophotometer (Thermo Scientific).

### Apoptosis

The Annexin V-FITC/PI apoptosis detection kit was purchased from Calbiochem (La Jolla, CA, USA). BRL cells were dispersed by 0.25% trypsin and 1–5×10^5^ cells were collected and washed twice with phosphate buffer (pH 7.4). Cells were suspended in 500 μl Annexin V binding buffer and mixed with 5 μl Annexin V binding buffer and 5 μl propidium iodide, and incubated for 10 min in the dark at room temperature. Cell apoptosis was detected by FCM (Cytomics FC500; Beckman-Coulter, Miami, FL, USA; Ex=488 nm, Em=530 nm).

### Mitochondrial membrane potential

BRL cells were cultured on coverslips prepositioned in 6-well plates overnight. After treatment, cells were processed with MitoCapture™ Apoptosis Detection kit (Calbiochem) and cultured at 37°C in a humidified incubator with 95% air and 5% CO_2_ for 20 min. MitoCapture is a cationic dye that exists as a polymer in the mitochondria of normal cells and produces a red fluorescence, or as a green fluorescent monomer in the cytoplasm of apoptotic cells. Results were recorded by fluorescence microscope (Eclipse 90i; Nikon) at x400 magnification.

### Lactate dehydrogenase (LDH) release assay

After treatment, BRL-cell medium was collected by centrifugation at 850 × g for 3 min and stored at −70°C. Samples were thawed and incubated at 37°C for 10 min. The tested sample (200 μl) was added to each tube. The absorbance of the resulting solution at 450 nm was measured with an automatic biochemical analyzer (DXC600; Beckman-Coulter). LDH activity was calculated against a sodium pyruvate calibration curve.

### Statistical analysis

Results were expressed as mean ± SD. Comparison between more than two groups was performed by one-way ANOVA and Student Newman-Keuls test. P<0.05 was considered to indicate a statistically significant result.

## Results

### LPS treatment increases CBS and H_2_S synthesis in BRL cells

**Figure 1:**
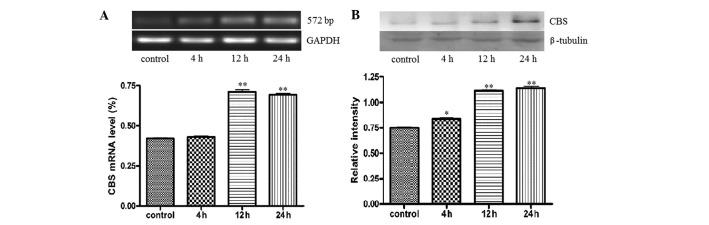
Alteration of endogenous CBS expression in LPS-treated BRL cells. BRL cells were treated with 10 μg/ml LPS and collected at the indicated times. (A) RT-PCR showing CBS mRNA. (B) Western blot analysis showing CBS protein. Data represent 3 independent experiments. ^*^P<0.05, ^**^P<0.01 vs. control. CBS, cystathionine β-synthase; LPS, lipopolysaccharide; RT-PCR, reverse transcription-polymerase chain reaction.

**Figure 2:**
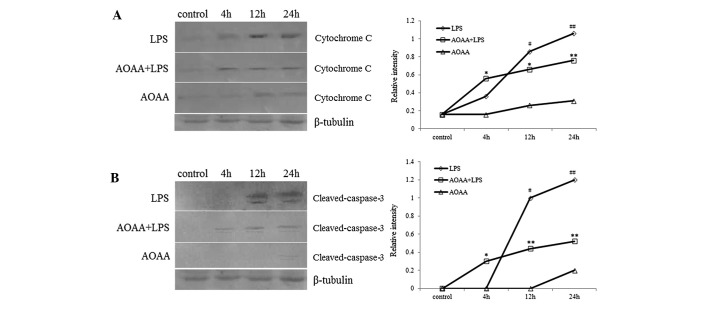
Western blot analysis of cytochrome *c* and cleaved caspase-3 in BRL cells after LPS-induced apoptosis. Cells were pretreated with or without the CBS inhibitor AOAA (3 mM) for 20 min before addition of LPS (10 μg/ml). At the indicated times, cells were collected for western blot analysis. (A) Variation in cytochrome *c* expression. (B) Alternation of cleaved caspase-3 protein. Data are representative of 3 independent experiments. ^#^P<0.05, ^##^P<0.01 vs. control; *P<0.05, ^**^P<0.01 vs. LPS group. CBS, cystathionine β-synthase; LPS, lipopolysaccharide; AOAA, aminooxyacetic acid.

**Figure 3:**
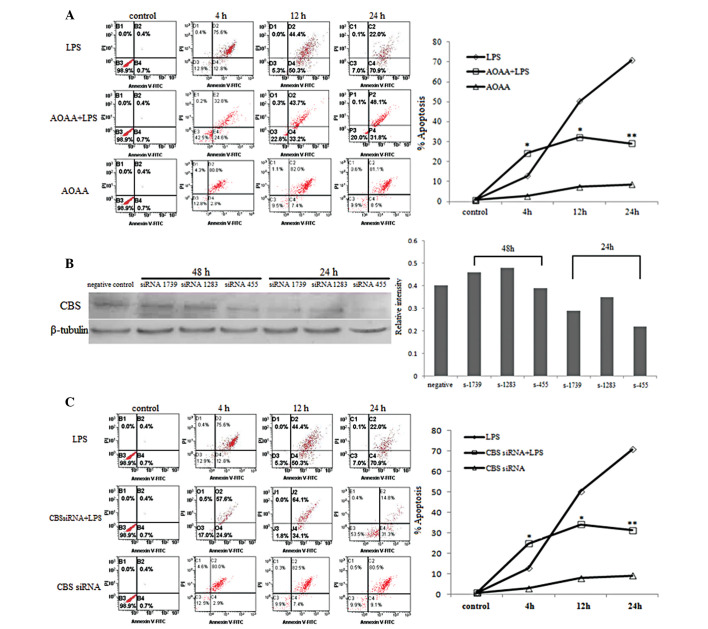
Effects of endogenous CBS on the apoptosis rates in BRL cells. (A) Apoptosis of BRL cells detected by FCM. Cells were pretreated with or without the CBS inhibitor AOAA (3 mM) for 20 min before the addition of LPS (10 μg/ml). At the indicated time, cells were collected for FCM. (B) Western blot analysis of endogenous CBS in cells treated with CBS siRNA. After transfection (24 and 48 h) with 3 different siRNAs, BRL cells were subjected to western blot analysis to detect CBS protein to determine the most efficient siRNA sequence and response time. (C) Apoptosis rate of BRL cells by FCM. After transfection with CBS siRNA, cells were treated with 10 μg/ml LPS for the indicated time. Data are representative of 3 independent experiments. ^*^P<0.05, ^**^P<0.01 vs. LPS group. CBS, cystathionine β-synthase; LPS, lipopolysaccharide; FCM, flow cytometry; siRNA, small interference RNA; AOAA, aminooxyacetic acid.

**Figure 4:**
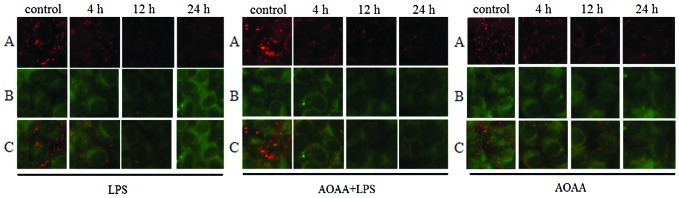
Variation in mitochondrial membrane potential in LPS-induced BRL cell apoptosis by fluorescence microscopy. BRL cells were cultured on coverslips prepositioned in 6-well plates overnight. Magnification, x400. (A) Red fluorescence is the polymer dye. (B) Green fluorescence is the monomer dye. (C) Overlap of A and B. LPS, lipopolysaccharide; AOAA, aminooxyacetic acid.

The expression of CBS was measured in BRL cells treated with 10 μg/ml LPS, the main ingredient in bacterial endotoxin, which was used to injure BRL cells. RT-PCR demonstrated that stimulation with LPS increased CBS mRNA over time from 4 to 24 h (P<0.05; Fig. 1A). Western blot analysis showed that CBS protein in BRL cells was stimulated by LPS, increasing significantly (P<0.05; Fig. 1B). The H_2_S production of BRL cells increased markedly (P<0.01; Table I). We used LDH as a common index for cell damage and observed that LDH in the culture medium significantly increased from 4 to 24 h after LPS treatment (P<0.05; Table II). In the early stages of apoptosis, mitochondrial cytochrome *c* can be detected in the cytoplasm. Western blot analyses for cytoplasmic proteins indicated that cytochrome *c* appeared at 4 h and markedly increased at 12 h after LPS treatment (P<0.05; Fig. 2A). Cleaved forms of caspase-3, indicating the activated form, appeared at 12 h and increased at 24 h after LPS treatment (P<0.05; Fig. 2B). FCM detection showed that apoptosis of BRL cells increased gradually from 4 to 24 h after LPS incubation (P<0.05; Table III and Fig. 3A). By contrast, variation in the mitochondrial membrane potential decreased from 4 to 24 h (P<0.05; Fig. 4A).

### LPS-induced CBS-H_2_S synthesis is inhibited by AOAA or CBS siRNA

BRL cells were pretreated with the CBS inhibitor AOAA (3 mM) for 20 min prior to the addition of 10 μg/ml LPS. At 4, 12 and 24 h, production of H_2_S in the AOAA+LPS group decreased significantly compared to the LPS alone group (P<0.05; Table I) and LDH in the culture supernatant of the AOAA+LPS group increased markedly at 12 h (P<0.01; Table II). Western blot analyses revealed that intracytoplasmic cytochrome *c* clearly increased in the AOAA+LPS group at 4 h but decreased at 12 h after LPS treatment compared to the LPS alone group (P<0.05; Fig. 2A). Although the level of cleaved caspase-3 fragments in the AOAA+LPS group was higher than in the LPS group at 4 h, LPS-induced detection of the cleaved caspase-3 fragment was strongly attenuated by AOAA at both 12 and 24 h (P<0.01; Fig. 2B). Detection by FCM indicated that apoptosis in the AOAA+LPS group increased at 4 h and decreased at 12 and 24 h compared to the LPS alone group (P<0.05; Table III and Fig. 3A). The variation in mitochondrial membrane potential in the AOAA+LPS group revealed a trend opposite to apoptosis compared to the LPS group (Fig. 4).

After the transfection with CBS siRNA, the addition of LPS to BRL cell cultures resulted in a significant decrease in H_2_S production compared to the LPS alone group (P<0.05; Table I). Apoptosis as detected by FCM increased in the CBS siRNA+LPS group relative to the LPS group at 4 h (P<0.05; Table III and Fig. 3C). However, apoptosis in the CBS siRNA+LPS group decreased significantly at 12 and 24 h compared to the LPS group (P<0.05; Table III and Fig. 3C).

### The effect of AOAA on BRL cells

When the CBS inhibitor AOAA was added to BRL cells at 3 mM, synthesis of H_2_S decreased over time (P<0.05; Table I). A similar affect was observed with transfection of BRL cells with CBS siRNA; CBS downregulation markedly decreased the level of H_2_S in BRL cells (P<0.05; Table I). LDH in the culture supernatant of BRL cells increased in the presence of AOAA (P<0.05; Table II), but western blot analyses showed that AOAA treatment did not alter the levels of intracytoplasmic cytochrome *c* or cleaved caspase-3 (Fig. 2), suggesting that AOAA had a minimal affect on BRL cell apoptosis. AOAA had little effect on apoptosis or MMP. These results demonstrate that LPS-induced apoptosis of BRL cells could be blocked by the CBS inhibitor AOAA.

## Discussion

H_2_S, named the third gaseous transmitter following nitric oxide and carbon monoxide, may be trans-membrane transported in a receptor-independent manner and activate various cellular targets (17). Particularly in the treatment of inflammation and ischemia-reperfusion injury, the enzyme CSE and H_2_S are an attractive pharmacological agent (9,18,19). As the primary H_2_S-generating organ *in vivo*, liver possesses the enzymes CSE and CBS (4). However, the enzyme CSE has been given more attention for its involvement in physiological and pathological conditions (20–24). Whether the CBS-H_2_S synthesis plays an important role in the modulation of hepatocyte apoptosis remains unknown.

In the present study, we treated the hepatic cell line BRL with LPS to generate an acute injury model of hepatocytes with the aim of observing the effect of the endogenous CBS-H_2_S synthesis on inflammatory lesions of hepatocytes. Our results indicate that CBS exists in the rat hepatic cell line BRL, as previously described (2). LPS treatment results in the upregulation of CBS mRNA and protein in BRL cells, with a corresponding increase in total H_2_S production. The apoptosis of BRL cells detected by FCM increased over time with a corresponding decrease in MMP. The appearance of cytochrome *c* in the cytoplasm increased and caspase-3 was activated by LPS treatment. The addition of the CBS inhibitor AOAA or transfection with CBS siRNA prior to LPS treatment in BRL cells resulted in an increase in cell apoptosis but no significant change in total H_2_S production at 4 h compared to an LPS alone control. However, the apoptosis of BRL cells decreased at 12 h and the H_2_S production also decreased, suggesting that endogenous CBS has short-term anti-apoptosis effects, and promotes apoptosis later. A possible explanation is CSE, another main endogenous enzyme involved in H_2_S generation (22,23). We hypothesize that since AOAA or CBS siRNA does not inhibit the function of endogenous CSE, H_2_S synthesis continued, and no significant change in total H_2_S production was observed between the AOAA+LPS and LPS alone groups. However, over time, endogenous CSE could not continue to enhance H_2_S synthesis. A similar tendency was observed with cytochrome *c* and cleaved caspase-3. Initially, cytoplasmic cytochrome *c* was clearly enhanced in BRL cells pretreated with AOAA prior to the addition of LPS, however, the expression then decreased compared to cells treated with LPS alone. At 4 h, cleaved caspase-3 appeared, suggesting activation, although the amount of cleaved caspase-3 was less than that in cells stimulated by LPS alone after 12 h.

In conclusion, our results indicate that endogenous CBS-H_2_S synthesis in BRL cells may regulate apoptosis induced by LPS, partly by involving the mitochondrial pathway. The course of regulation is complex and may be anti-apoptotic in the short-term but pro-apoptotic in the long-term. These results may provide references for the research and development of clinical treatments. The specific mechanism of the regulation and interaction of endogenous H_2_S synthesis requires further investigation.

## Figures and Tables

**Table I:** Alteration of H_2_S production in BRL cells (μmol/l).

Groups	Control	4 h	12 h	24 h
LPS group	26.19±4.93	36.23±4.23	46.73±3.44^a^	58.23±2.52^a^
AOAA+LPS group	26.17±4.57	24.33±2.43	19.25±2.24^b^	16.98±4.02^c^
AOAA group	26.18±4.45	24.74±3.98	18.66±4.31	13.42±2.45^d^
CBS siRNA+LPS group	26.17±4.77	24.26±3.23	19.77±2.27^b^	16.22±3.55^c^
CBS siRNA group	26.19±4.66	24.01±4.11	18.46±3.37	13.13±3.01^d^

a P<0.01 vs. control group,

b P<0.05 and

c P<0.01 vs. LPS group,

d P<0.05 vs. control group. LPS, lipopolysaccharide; AOAA, aminooxyacetic acid; AOAA+LPS, pretreated with AOAA prior to the addition of LPS in BRL cells; CBS siRNA, transfection of CBS siRNA in BRL cells; CBS siRNA+LPS, pretreated with CBS siRNA prior to the addition of LPS in BRL cells; CBS, cystathionine β-synthase; siRNA, small interference RNA.

**Table II:** Alteration of culture supernatant LDH (U/l).

Groups	Control	4 h	12 h	24 h
LPS group	40.33±2.08	52.27±10.90	76.20±3.27^a,b^	118.17±23.58^b,c^
AOAA+LPS group	40.31±2.11	51.33±2.31	113.67±5.69^c,d^	186.33±6.43^c,d^
AOAA group	40.34±2.01	46.01±7.01	65.00±5.29^a,b^	100.33±2.52^c,d^
CBS siRNA+LPS group	40.28±1.97	52.21±5.27	109.16±3.42^c,d^	182.41±5.27^c,d^
CBS siRNA group	40.31±2.07	44.21±3.23	49.12±3.42	56.22±5.55

a P<0.05 vs. control group,

b P<0.05 vs. 4 h group,

c P<0.01 vs. control group,

d P<0.01 vs. 4 h group. LPS, lipopolysaccharide; AOAA, aminooxyacetic acid; AOAA+LPS, pretreated with AOAA prior to the addition of LPS in BRL cells; CBS siRNA, transfection of CBS siRNA in BRL cells; CBS siRNA+LPS, pretreated with CBS siRNA prior to the addition of LPS in BRL cells. LDH, lactate dehydrogenase; CBS, cystathionine β-synthase; siRNA, small interference RNA.

**Table III:** Variation of apoptosis rate in BRL cells (%).

Groups	Control	4 h	12 h	24 h
LPS group	0.7±0.1	12.8±0.2^a^	50.3±0.5^b^	70.9±0.3^b^
AOAA+LPS group	0.7±0.1	24.6±0.3^c^	33.2±0.5^c^	31.8±0.4^d^
AOAA group	0.7±0.1	2.8±0.2	7.4±0.8	8.5±0.6
CBS siRNA+LPS group	0.7±0.1	24.9±0.7^c^	34.1±0.2^c^	31.3±0.2^d^
CBS siRNA group	0.7±0.1	2.9±0.1	7.9±0.4	9.1±0.3

a P<0.05 and

b P<0.01 vs. control group;

c P<0.05 and

d P<0.01 vs. LPS group. LPS, lipopolysaccharide; AOAA, aminooxyacetic acid; AOAA+LPS, pretreated with AOAA prior to the addition of LPS in BRL cells; CBS siRNA, transfection of CBS siRNA in BRL cells; CBS siRNA+LPS, pretreated with CBS siRNA prior to the addition of LPS in BRL cells. CBS, cystathionine β-synthase; siRNA, small interference RNA.
